# Bovine trypanosomosis: changes in parasitemia and packed cell volume in dry and wet seasons at Gidami District, Oromia Regional State, western Ethiopia

**DOI:** 10.1186/s13028-017-0327-7

**Published:** 2017-09-11

**Authors:** Efrem Degneh, Workineh Shibeshi, Getachew Terefe, Kaleab Asres, Hagos Ashenafi

**Affiliations:** 1 Department of Pathology and Parasitology, College of Veterinary Medicine and Agriculture, Addis Ababa University, P.O.Box 34, Debre Zeit, Ethiopia; 2 Department of Pharmacology and Clinical Pharmacy, School of Pharmacy, College of Health Sciences, Addis Ababa University, P.O.Box 9086, Addis Ababa, Ethiopia; 3 Department of Pharmaceutical Chemistry and Pharmacognosy, School of Pharmacy, College of Health Sciences, Addis Ababa University, P.O.Box 9086, Addis Ababa, Ethiopia

**Keywords:** Agro-ecology, Gidami, PCV, Prevalence, Season, Bovine trypanosomosis, Western Ethiopia

## Abstract

**Background:**

Animal trypanosomosis is one of the major disease problems affecting agricultural productivity in Ethiopia. The impact of the disease is believed to vary with season and agro-ecologies in line with fly vector distribution. A cross-sectional study on bovine trypanosomosis was conducted from November 2015 to June 2016, in seven selected villages of Gidami district, Oromia Regional State, western Ethiopia. A total of 930 blood samples were collected and subjected to parasitological and hematological analysis.

**Result:**

The overall prevalence of bovine trypanosomosis was 14.1%. The seasonal prevalence shows 9.06% in early dry and 18.4% in early rainy seasons. Three trypanosome species, *Trypanosoma congolense, Trypanosoma vivax* and *Trypanosoma brucei* were identified in the examined animals. *T. congolense* followed by *T. vivax* were the predominant species (respectively 59.0 and 35.9% in early dry season and 62.0 and 22.8% in early rainy season). The prevalence of *T. vivax* remained similar in both early dry and early rainy seasons in both lowland and midland agroecologies whereas *T. congolense* was more dominant in the lowland area in both seasons compared to mid land study sites. The disease was more prevalent in lowland (23.9%) compared to midland (11.1%) during early rainy season (P < 0.001) whereas no significant difference was observed between the two agroecologies during early dry season (P = 0.165). Packed cell volume (PCV) was much lower in parasitemic animals than in aparasitemic cattle whereas the mean PCV value for parasitemic animals (20.36%; 95% CI 19.56 to 21.16) in early dry season was similar to values in early rainy season (20.46%, 95% CI 18.84 to 21.08%). A similar situation was noticed for animals in both low land and mid land study sites.

**Conclusion:**

Overall, the detection of trypanosomes in blood was significantly affected by agro-ecology, season and body condition of the animals. Special emphasis should be given to integrated trypanosomosis management in early rainy months where fly population is believed to start increasing.

## Background

African animal trypanosomosis (AAT) is the most important constraint to livestock production in tropical Africa [1–3], and considered as a threat to poverty alleviation programs in the continent [4]. The disease is widely distributed with about 50 million heads of cattle and other livestock species being at risk [5]. It is cyclically transmitted by tsetse flies [6] and mechanically by a number of biting flies [7]. Animal trypanosomosis is most important in cattle, but can also cause serious losses in camels, equines, goats and sheep. In Africa the annual direct and indirect losses due to this disease for livestock are estimated at 4.5 billion USD [8].

Ethiopia is believed to have the largest livestock population in Africa, which is currently estimated to be 54 million cattle, 25.5 million sheep and 24.1 million goats [9]. However, the livestock industry of the country is suffering from debilitating diseases such as trypanosomosis with approximately 15% of all arable land or 220,000 km^2^ area infested with tsetse flies [10]. Morbidity and mortality losses from ruminant livestock alone are estimated to be USD 200 million [11].

Although a number of studies have reported the prevalence of the disease in many places of the country [12–14], data on the seasonal dynamics of the problem and associated risk factors is scanty for the Gidami district of the Oromia Regional State, western Ethiopia. The Gidami district is one of the districts richly endowed with livestock resources but is ravaged by animal trypanosomosis (District office of Livestock and Fisheries: personal communication). Therefore, the present study was aimed at assessing the prevalences of bovine trypanosomosis and associated risk factors in early dry and early rainy seasons, in order to provide baseline data that can be used in planning and implementation of disease control program in this area.

## Methods

### Study area

**Fig. 1:**
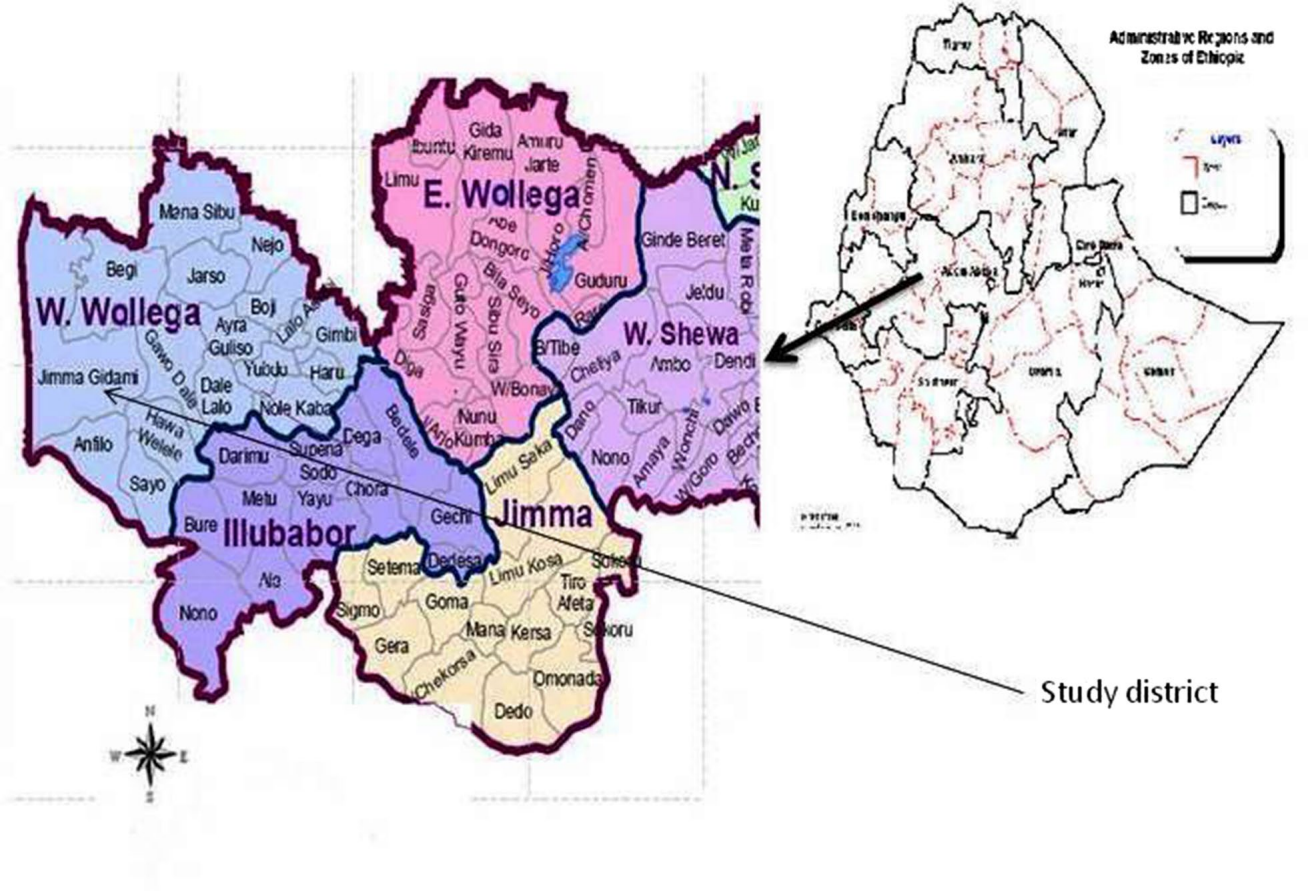
Map showing the location of the study area in western Ethiopia

This study was conducted from November 2015 to June 2016 in the Gidami District of West/Kellem Wollega Zone of the Oromia Regional State (western Ethiopia). The Gidami district is located 670 km to the west of the capital, Addis Ababa (Fig. 1). The area has two distinct seasons: a dry season extending from November to May; and a wet season which extends from June to September. It has a total area of 219,641 hectares. The altitude of the area ranges from 1200 to 2200 m above sea level. Based on the altitude the area is subdivided into three climatic zones: highland (8%), midland (75%) and lowland (17%). The mean annual rainfall and temperature ranges of the area are 1200 to 2000 mm and 15 to 25 °C respectively. Mixed crop-livestock farming is the main source of livelihoods. The vegetation cover is dominated by savanna grassland, forest, riverine and bush lands. Wild games like buffalos, bush pig, warthog, lions, antelopes, leopard, hyena and monkeys are commonly found in the area. The livestock population of the district has been estimated to be around 73,000 cattle, 47,000 sheep, 24,000 goats, 12,000 equines and 140,000 chickens [9]. Communal grazing without any supplementary feeding is the major livestock husbandry system throughout the year.

### Study design and sampling methods

A cross-sectional study was conducted to estimate the prevalence of bovine trypanosomosis in the study area. Seven peasant associations were selected purposively based on livestock population and accessibility from two agro-climatic zones: four from lowland (<1600 m above sea level) and the rest three from midland (>1600 m above sea level). Animals were bled during early dry (November and December) and early rainy (May and June) seasons of the year. A systematic random sampling technique was employed to sample every other individual animal caught at communal grazing points of each village. The sample size was determined based on the expected prevalence of 16.9% as previously reported by Kebede [15] for the nearby district, Sayo Nole in West Wollega, and absolute desired precision of 5% at 95% confidence level as described by Thrusfield [16]. Accordingly, 215 animals were required to be sampled in each study season (total = 430). However, because of adequate availability of animals, this number was increased to 930 to improve the precision. Accordingly, a total of 514 animals from the lowland area (230 and 284 in dry and wet season respectively) and 416 animals from the midland areas (200 and 216 in dry and wet season respectively) were sampled.

### Study population

The study population consisted of all zebu cattle above 1 year of age, which were usually kept under an extensive husbandry system. A total of 930 animals (500 males and 430 females) were systematically selected in early dry and early rainy seasons. The age of animals was determined by dentition [17] and conventionally categorized as young (1 to 3 years) and adult (>3 years). The body condition score of animals was recorded by classifying animals into three groups as good, medium, and poor based on the appearance of ribs and vertebral spinous processes [18].

### Blood sampling and examination

Blood samples were collected by puncturing the superficial ear vein of each animal into heparinized capillary tubes and centrifuged immediately in a hematocrit centrifuge. Packed cell volume (PCV) was measured for each sample. Animals with PCV less than 24% were considered to be anemic [19, 20]. The contents of the capillary tubes (including about 1 mm above and below the buffy coat) were examined using buffy coat technique to reveal trypanosomes under 40× magnifications under the light microscope [21–23]. From trypanosome positive samples thin blood smears were made and stained with Giemsa for species identification by light microscopy. The trypanosome species were distinguished using their size, position of the kinetoplast, presence of undulating membranes and length of the free flagella according to [20, 24].

### Data analysis

Data collected from each study animal and laboratory analyses were entered into a Microsoft excel spreadsheet and un-coded. SPSS version 20 was used for the analysis and interpretation of the data. The prevalence of trypanosomosis was calculated as the number of infected individuals divided by the number of individuals examined and multiplied by 100. The difference in prevalence between altitude, season, sex, age and body condition score was compared by Chi square test. Student’s t-test was used to compare the mean PCVs between parasitemic and aparasitemic animals, among seasons and agroecologies. Generally, P < 0.05 was considered to be statistically significant.

## Results

### Parasitological findings

**Fig. 2:**
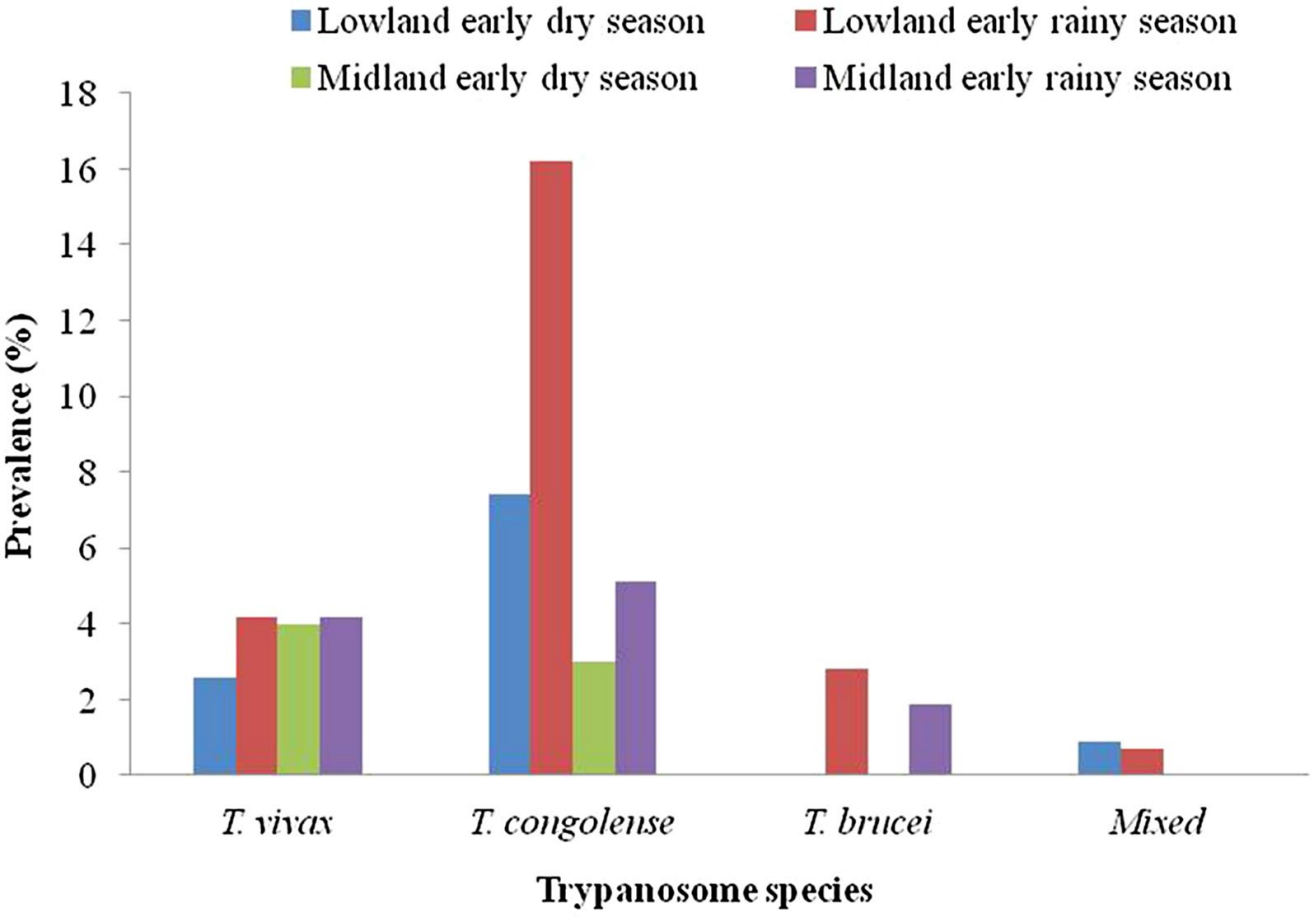
*Trypanosome* species identified during the study period in Gidami District, western Ethiopia

Out of 930 examined cattle, 131 were positive for trypanosomosis using the buffy coat technique with an overall prevalence of 14.08% (95% CI 11.9 to 16.5%). The prevalence was significantly higher in low land than in mid land, during early rainy season than in early dry season and in animals with poor and moderate body condition compared to those with good body condition (P < 0.001). Similarly, the prevalence of the infection in poor body conditioned animals was significantly higher than in animals with medium body condition (Table 1). There was a notable increase in the prevalence of bovine trypanosomes in low land areas during early rainy season as compared to the mid land (P < 0.001) whereas no significant difference was detected between low land and mid land areas during early dry season (P = 0.165) (Table 2). Three trypanosome species, *T. congolense, T. vivax* and *T. brucei* were identified in the study animals. *T. congolense* was the predominant species (59 and 62%) followed by *T. vivax* (35.9 and 22.8%) in early dry and early rainy seasons respectively (Fig. 2).

**Table 1:** Prevalence of bovine trypanosomosis with different potential risk factors in Gidami district, Oromia Regional State, western Ethiopia

Potential risk factors	No of animals	Prevalence (%)	95% CI	χ^2^ value	P-value
Altitude					
Low land	514	18.09	14.76 to 21.42	15.24	0.0001
Middle land	416	9.13	6.36 to 11.9		
Season					
Early dry	430	9.07	6.36 to 11.78	16.61	<0.0001
Early rainy	500	18.4	15 to 21.80		
Body condition					
Poor	294	27.9	22.77 to 33.03	39.16 14.95	<0.0001 0.0001
Medium	458	10.3	7.52 to 13.08		
Good	177	1.1	−0.44 to 2.64		
Sex					
Male	500	14.4	11.32 to 17.48	0.09	0.766
Female	430	13.72	10.47 to 16.97		
Age					
1–3 years	291	11.34	7.7 to 14.98	2.64	0.104
>3 years	639	15.34	11.55 to 16.73

**Table 2:** Prevalence of bovine trypanosomosis during early rainy and early dry seasons in low land and midland areas of Gidami district, western Ethiopia

Season	No. of animals	Prevalence (%)	95% CI	χ^2^ value	P-value
Early rainy season					0.0003
Low land	284	23.9	18.94 to 28.86	13.36	
Mid land	216	11.11	6.92 to 15.30		
Early dry season					0.1649
Low land	230	10.86	6.84 to 14.88	1.93	
Mid land	200	7	3.46 to 10.54

### Packed cell volume

Statistical analysis of the PCV values indicated that the overall mean PCV was significantly lower for trypanosomosis positive animals (20.48%, 95% CI 19.97 to 20.99%) compared with that of negative animals (25.77%, 95% CI 25.48 to 26.07%) irrespective of season of sampling and agro-ecology (P < 0.001). On the other hand, there was no notable difference between mean PCVs of trypanosomosis positive animals when lowland and midland agroecologies (P = 0.331) as well as early rainy and early dry seasons (P = 0.379) were compared (Table 3). Strong association between prevalence of trypanosomosis and anemia (expressed in terms of reduction in PCV below 25%) was observed (P < 0.001) in both sampling seasons and agroecologies (Table 4). Among the trypanosomosis positive animals, 92.3 and 91.3% fall in anemic category in early dry and early rainy seasons respectively while 90.3 and 94.7% of them were anemic respectively in lowland and midland sampling sites.

**Table 3:** Comparison of PCV of parasitologically positive animals according to sampling season and agro-ecology

Risk factors	Category	No. of parasitemic animals	% PCV	95% CI	*t.* value	P-value
Season	Early dry	39	20.36	19.53 to 21.19	−0.308	0.379
	Early rainy	92	20.53	19.89 to 21.17		
Agro-ecology	Low land	93	20.41	19.81 to 21.00	−0.439	0.331
	Mid land	38	20.66	19.64 to 21.68

**Table 4:** Comparison of trypanosome prevalence between anemic and non-anemic cattle during early dry and early rainy seasons from lowland and midland study areas

Category	Anemia status^a^	No. of animals examined	% positive animals	95% CI	*χ* ^*2*^ value	*P*-value
Early dry	Anemic	179	20.10	14.20 to 26.0	36.51	<0.0001
	Non-anemic	251	1.20	0.0 to 2.5		
Early rainy	Anemic	274	30.66	25.2 to 36.1	60.03	<0.0001
	Non-anemic	226	3.54	1.1 to 5.9		
Low land	Anemic	271	31.00	25.5 to 36.5	67.87	<0.0001
	Non-anemic	243	3.70	1.20 to 5.50		
Mid land	Anemic	182	19.78	14.00 to 25.60	44.178	<0.0001
	Non-anemic	234	0.85	0.00 to 2.00		

^a^Anemic: PCV ≤24%, non-anemic: PCV >24%

## Discussion

The present study revealed that trypanosomosis is widespread in Gidami District with an overall prevalence of 14.08% (95% CI 11.9 to 16.5%). This result was slightly lower than the findings of Kebede [15] who reported an overall prevalence of 16.9% in the nearby Sayo Nole District. The fact that there were no vector control intervention practices in the study area during the study period suggest that any reduction could be attributed to difference in season of sampling and/or changes in ecological/climatic conditions that affect vector fly density/distribution overtime [25]. Higher prevalence values were also reported by Cherenet et al. [26] and Mulaw et al. [27] respectively with 25.7 and 28.1% proportions from different parts of Ethiopia. On the other hand, the prevalence reported here is in close agreement with the report of Feyissa et al. [28] in selected Villages of Humbo District, southern Ethiopia (14.2%) and higher than the 2.86% reported by Biyazen et al. [29]. Such variations may exist because of differences in agro-ecology, sampling season, vector infection rate, animal susceptibility and practice of trypanocidal drug use and fly control operations which may obviously impact on epidemiological situations of the disease [30–32].

The findings in the present study is in agreement with the fact that the most pathogenic trypanosome species for cattle, i.e., *T. congolense* and *T. vivax*, are abundant in most parts of western Ethiopia ([10, 27], in Ethiopia in general as well as in other parts of Africa [33–35]. The high proportion of *T. congolense* is similar with the previous report of Duguma et al. [36] in south-western Ethiopia (76%), Ameen et al. [37] in Ogbomoso Area of Oyo State, Nigeria who reported only *T. congolense* infection and Dawud and Molalegne [38] at Mao-Komo district of Benshangul Gumuz regional state (63.2%). The high ratio of *T. congolense* may be ascribed to the more efficient transmission of this species by major cyclical vectors (tsetse flies) than *T. vivax* in tsetse infested areas [33].

Although the rate of infection with trypanosomes was high in both study periods, it was significantly higher at the beginning of the rainy season, particularly with *T. congolense*. This agrees with the reports of Majekodunmi et al. [31]. Although fly burden was not assessed in this study, the high incidence of trypanosome infections at the beginning of the rainy season may be explained by the increasing density of tsetse and other biting flies during this time of the year [39]. It is also possible that the cumulative effect of feed shortage during the dry season preceding the sampling may have reduced immunity of the animals while favoring higher infection prevalence. Holmes et al. [40] and Katunguka-Rwakishaya et al. [41] reported that high protein intake reduces the pathologic effect of trypanosomosis and enhances recovery following treatment with trypanocidal drugs. On the other hand, our finding does not agree with the report of Ameen et al. [37] which indicated higher rate of *T. congolense* infection in dry season than in wet season which probably is explained by the reason given by the authors. They suggested that presence of few ponds in the dry season might have forced the animals to come close together and also created a favorable ground for the tsetse flies.

The prevalence of bovine trypanosomosis is also known to be affected by agro-ecological conditions such as altitude [42]. In this study, the prevalence of trypanosomosis was significantly higher in lowland areas compared to those in mid-land study sites during the early rainy season. This suggests the possible development of optimum vegetation, temperature and humidity favorable for tsetse fly breeding and survival [35]. This finding agrees with other studies done elsewhere [43]. The absence of difference in prevalence of the disease during early dry season may suggest that the two agro-ecologies did have adequate vegetation for the animals to graze and exposure to fly infestation have started declining.

Body condition of the cattle was another factor that has shown strong association with trypanosomosis prevalence. Animals with poor body condition were found more affected by trypanosomes than other cattle with good body condition. This finding is in line with previous reports [44–47]. This might be attributed to reduced resistance of those animals having poor body condition or related to the progressive weight loss arising from debilitating nature of the disease itself [48].

In line with other previous studies on bovine trypanosomosis [42, 44, 49, 50], anemia was significantly more severe in trypanosome infected cattle (as evidenced by lower mean PCV) compared to trypanosomosis negative animals. This is further supported by the fact that majority of trypanosomosis positive animals had PCV values lower than 25% and the non-anemic category had very low proportion of trypanosome positive cases. On the other hand, the majority of the animals classified as anemic had no detectable trypanosomes suggesting that other anemia causing factors such as poor nutrition, helminthiasis etc. could be responsible for the reduction in PCV. In this regards, our findings agree with the reports in other previous studies [51–53]. It may also be partly due to the low sensitivity of parasitological diagnostic method used in this study [49, 54, 55] which has resulted in some trypanosome positive animals with lower PCV to be wrongly categorized as negative for trypanosomes. Trypanosomes become very difficult to detect when the parasitemia is lower than 60 trypanosomes/mL blood [56, 57].

## Conclusions

The current study indicated that the prevalence of trypanosomosis is significant to the level that it can limit livestock production in the Gidami District of Ethiopia. Two pathogenic species, *T. congolense* and *T. vivax* were mainly responsible for the disease in the study area. Significant variation in prevalence was also observed between seasons, agro-ecology and animal body condition scores. Anemia was characteristic of the infection in both lowland and mid land agroecologies irrespective of the sampling season. The situation warrants the initiation and intensification of tsetse fly control activities especially in the early wet season where *T. congolense* is most dominant.
